# Supplementary material for: Do selfies make women look slimmer? The effect of viewing angle on aesthetic and weight judgments of women’s bodies
Source: PLoS One. 2023 Oct 11;18(10):e0291987. doi: 10.1371/journal.pone.0291987 (PMC10566732; doi:10.1371/journal.pone.0291987)
Supplement: Attachment — Submitted filename: PONE-D-22-21961R1_ST.pdf [file pone.0291987.s002.pdf]

Do selfies make women look slimmer? the effect of viewing angle on aesthetic and weight judgments of women's bodies (PONE-D-22-21961R1)

Revision Number 1

Sofia Tagini

I appreciate the effort the Authors made in revising the manuscript according to the Reviewers' suggestions. On my side, I am quite satisfied with how the Authors addressed my previous comments: I believe the manuscript has highly benefitted from revisions. However, I may ask for some further revisions, of moderate entity, which I believe would further strength the paper.

Overall, the new structure greatly improved readability. However, I may suggest doing an additional effort: in the description of each experiment there are elements which are common to the three of them (either repeated in each paragraph or reported only in experiment one and then referenced below).

Detecting all common factors to experiment one-to-three and illustrating these shared aspects/procedures (analyses, stimuli, etc.) before describing each single experiment would further simplify the manuscript structure and reduce redundancy, improving the manuscript intelligibility. For example, the description of the EDE-Q, which is reported in experiment one methods and then referenced two times in the methods of experiment two and three, may be described only once before explaining the singularities of each experiment, clarifying that the same measure was included in experiment one-to-three.

Lines 113-115: I strongly suggest the Authors to revised this sentence, which in the present wording suggests that women intentionally try to appear submissive to the purpose of attracting dating partners (indeed, the paper mentioned by the Authors specifically focused on selfies posted on Tinder). Beyond resulting absolutely disqualifying for women, this was not the interpretation given by Sedgewick and colleagues. Briefly reading their work, I understand that males and females tend to prefer poses in which they seem higher/smaller (respectively) according to (possibly covert) expectations about the height preferred by each counterpart. Then, the authors hypothesized why women may preferred height men and men might prefer smaller women (i.e., suggesting that men may prefer smaller women because they (the men) associate smaller women to faithfulness and subordination). In other words, women might try to appear smaller (consciously or not) because this is men's preferred height, but there is no evidence - at least in the mentioned work - that women intend to seem submissive. Also, I would specify that the mentioned work only referred to pictures posted for dating purposes; in fact, the Authors may find interesting the more recent findings and considerations provided by Soranzo and Bruno (10.1371/journal.pone.0238588).

Lines 204/205 and 1060-1065: I agree that most of the literature on body-related topics focused on females only; however, I believe this fact is not a solid reason *per se* to include only women in the sample (in fact, it may encourage more studies on men!). What I mean, is that the Authors should clarify *why* considering women and men separately is necessary (which is the reason why this was frequently done in the literature on body representation) and why they specifically choosed to investigate females rather than men (e.g. are they more sensitive to social medial influence? Are they more disposed to body image issues?)

Line 214: Could the Authors clarify whether social media adverts were limited to students or none "external" participant answered the call?

Line 218: I renew my suggestion of providing sample size analyses after the explanation of the analyses planned. I am not familiar with R procedure (anyway, the software should be referenced), but sample size computations usually asked for a specification of the statistical method (e.g., f-tests, t-tests etc.). The Authors referred to "medium effect size" but effect sizes are computed and interpreted according to the statistical methods adopted: in the present form, the explanation of sample size calculation seems not grounded. Also, I suggest the Authors clarifying why they included more participants than the number identified. Sticking to a priori sample size calculation is a good research practice to avoid arguable "results seeking" by adding participants progressively. I am not insinuating at all that this was what the Authors did, but by clarifying the reason behind this choice may safeguard themselves.

Figure 1: I would suggest re-ordering the four pictures according to the order they are mentioned in the manuscript. Also, referring to the upper/lower part of the Figure 1 in the corresponding task may be more incisive.

Analyses

Line 297: I suggest reporting the analyses concerning the experimental task (i.e., attractiveness/weight judgments) first and then (e.g., before line 307) those relative to the EDE-Q: this order seems more congruent with the timing of tasks/questionnaire administrations and with the "hierarchy" study aims (i.e., as I understand the core aim is to compare judgments relative to different perspective, while the possible correlation with eating disorders is secondary). Also, as previously mentioned, I would report the analyses (which they seem the same in experiment one-to-three, before introducing each single experiment).

Anyway, concerning correlational analyses, I am not confident that normality should be checked for EDE-Q scores given the ordinal nature of Likert-scores. This is, indeed, only a matter of writing since the Authors used non-parametric correlational analyses.

In this regard, did the Authors checked for the actual achieved power of the correlational analyses? Since sample size was computed on pairwise comparisons, correlations may/may not be enough powered, possibly explaining why the Authors did not find certain expected results. The Authors may then consider to discuss this point among limitations. Similarly, did the Authors considered/analysed outliers? It is quite interesting that dependent variables in experiment one-to-three were all non-normal: outliers may be a related issue. Additionally, Authors may consider providing boxplots with participants single data point for each experiment, this may help them detecting outliers/extreme values and the reader to acknowledge data distribution.

Could the Authors provide the adopted alpha critical level for pairwise comparisons?

Line 299: Please, clarify how the dependent variable was computed. This wording suggests that the Authors got ten measures of weight/attractiveness relative to each perspective: were then these measures collapsed (how?) into one variable (one for weight/attractiveness) in each perspective?

Furthermore, the Authors may make explicit the purpose of parametric/non-parametric analyses: they reported that paired-sample t-tests/ Wilcoxon tests were used, but it is not clear to which purpose. I imagine the goal was comparing weight/attractiveness between the two perspective in each experiment: is that possible that this is indeed reported below at line 302-205? If this is the case, I strongly suggest moving this part before. Additionally, if the Authors would accomplish my suggestions of reporting common analyses before, here (and after) they might only report the hypothesis tested in this experiment (e.g., lines 302-303).

Table 1: I was wondering why the Authors collected participants' BMI, although it seems reasonable. Is this to ensure that participants were not overweight/underweight? If so, why this should be considered relative to the study purposes? Did the Authors compared participants' BMI and models' BMI?

Also, it may be useful and more informative to report the perspective compared near the number of the experiment.

Line 545: I wonder whether the Authors could support the hypothesis of a "bias against egocentric pictures" somehow with their results (please considered this more a curiosity, rather than a critical point). For instance, I would be curios to know whether the BMI was associated with the ratings of the egocentric pictures/or the preference for selfies (especially considering that there was no -the expected - correlation with eating disorders measures). In this regard, the Authors may consider that BMI seems associated to body size distorted judgments (e.g., see Tagini et al., 2021 https://doi.org/10.1007/s00221-021-06215-4)

Results

Overall, since many results are reported, I believe that summarizing tables may efficaciously help the reader to compare results across studies, at once.

Experiment Four

Line 734: As already mentioned, I suggest reporting sample size calculation after the analyses section since expected power is computed accordingly. In this regard, please specified that one-way "repeated measures" ANOVAs were used.

Line 743: Could the Authors clarify how many trials were included in each block (please, also in the previous experiments)? Was each combination of model/perspective repeated only one time (10 trials in each perspective)?

Line 772: Could the Authors specify why the Bayes factor was computed only in experiment four?

Line 778: I believe the figure here is in the wrong place

General Discussion

Lines 908-916: I found this point on the possible effect of personality traits associated to selfies a bit confusing. It seems that selfies are related to both “good” and “bad” attributes but it is not clear how these sides related to the study results, respectively. I would clarify the wording and the hypothesis proposed: indeed, it is quite interesting (and reasonable) that weight judgments rely more on perceptive bottom-up processing of visual information whereas attractiveness judgments may likely include also top-down components. Did the Authors try to correlate slimness and attractiveness judgments in each perspective?

Finally, I suggest a careful check for typos.
