# Supplementary material for: Do selfies make women look slimmer? The effect of viewing angle on aesthetic and weight judgments of women’s bodies
Source: PLoS One. 2023 Oct 11;18(10):e0291987. doi: 10.1371/journal.pone.0291987 (PMC10566732; doi:10.1371/journal.pone.0291987)
Supplement: Attachment — Submitted filename: Response to Reviewers.docx [file pone.0291987.s001.docx]

**Manuscript ID: PONE-D-22-21961: Response to Editor and Reviewers**

**"Do selfies make women look slimmer? the effect of viewing angle on aesthetic and weight judgments of womens’ bodies.”**

In addition to the quoted text (in italics) within the present document, the below changes can be found in the tracked changes version of the revised manuscript.

**Reviewer One Comments**

**1. The title of the MS should be reconsidered, imho. The main reason is that the authors only used female models and the title clearly suggests that results can be generalized. Moreover, the authors do not even discuss this point. My suggestion to solve this problem: Change the title so that the reader is aware of this fact or just add this to your limitations**

Many thanks for the useful comments regarding the title of the manuscript. We have changed the title as below to ensure that it more accurately reflects the findings of the study.

*"Do selfies make women look slimmer? the effect of viewing angle on aesthetic and weight judgments of women’s bodies.”*

In addition, we have also added justification for using a female only sample in the introduction Page 8

*“Given that much of the research into body satisfaction, use of social media, and attractiveness judgements has used predominantly female samples, we focused on women in this study.”*

As well as including this as a limitation in the discussion page 46

*“That only female participants took part in this study could also limit our findings somewhat. We focused on women’s aesthetic judgements as all of the models for the stimuli were women and we were interested in potential impact on social comparisons and body satisfaction. It may be that ratings of these stimuli from non-female participants would differ. Future research should consider whether there is an impact of model and/or participant gender on aesthetic judgements.”*

**2. Personally, I found it very interesting and convincing that past research (e.g., Schneider & Carbon 2017 as well as Schneider, Hecht & Carbon, 2012) did not use full-body selfies and that it might be important to combine faces + bodies since this could be more naturalistic (page 10 in your MS). Unfortunately, the authors limited their stimulus material to body only depictions. My suggestion: Make the (potential?) strength of your approach more clear. Otherwise, discuss why you did not follow your own argumentation (weaknesses).**

This useful suggestion enabled us to consider our decision to use only the bodies of models in the stimuli for the experiment. Given that research indicates that the body and face make independent contributions towards attractiveness, and that this study considered disordered eating and related body (dis)satisfaction, it seemed that including the faces of models might add a confounding variable. The models faces may have influenced participants judgements of the models bodies. This set of experiments focused on the aesthetic judgements of bodies specifically, and we have adapted the introduction to make this clearer (pages 6/7).

*“When we make aesthetic judgements, we typically look at bodies and faces together (Peters, Rhodes & Simmons, 2007). However, research suggests that face and body judgements do not interact when individuals make an overall attractiveness judgement; instead, both make significant independent contributions to overall attractiveness (Peters et al., 2007). For women, both face and body components influence overall attractiveness, which validates looking at attractiveness judgements of faces and bodies together and individually (Peters et al., 2007). Previous research indicates that visual perspective can influence aesthetic judgements of bodies; this work builds on those findings by adding selfie angles and specifically considering social media style images (Carey et al., 2019). Some research indicates that faces may provide more information used in attractiveness than bodies (Currie & Little, 2009). Therefore, as we are interested specifically in how social media style images influence feelings towards the body, we did not include models’ faces, as it may have resulted in participants rating the attractiveness of the model based more heavily on judgements of their faces as opposed to aesthetic judgements of the body.”*

We also added a passage about this to the discussion section, when considering limitations of the experiments (page 45).

*“However, in practice social media users more rarely see bodies depicted without faces (although this does happen, particularly through fitspiration accounts and content). This may have affected the ecological validity of this series of experiments, as we asked participants to make judgements of bodies without faces. Future research should consider whether similar effects as those found in this study are also found when stimuli are images of bodies with faces included in the image.”*

**The authors did use four viewing angles. I found it hard to understand why they used these perspectives. Is this approach derived from scientific research? Frontal (“allocentric”) is reasonable. However, why is the frontal depiction defined as a selfie? The authors should provide information why only this perspective can be seen as a selfie (in contrast to the “selfie-stick”-condition). There is scientific evidence that other perspectives also can be defined as selfies + have stronger impact on perceived body related factors (see e.g., Schneider & Carbon, 2017).**

We have now added a definition of a selfie to the introduction (page 4)

*“Selfies have been differentiated from traditional (allocentric) portraits due to the fact that they are taken by the subject of the picture (Schneider and Carbon, 2017). As such these images have a unique viewing perspective, which does not subscribe to fundamental principles traditionally applied to portrait photography (Bruno et al. 2013) (also see Yeh and Lin 2014).”*

And clarified that selfie-stick images are another type of selfie, that is aimed to modulate distance between the camera and the body (page 26).

*“Selfies are now not only taken by holding the camera away from you with your arm. As social media and platforms like Youtube developed, along with the tendency to take photos or videos from this perspective, other tools for capturing this kind of content were created. One such tool is the selfie-stick. These are commonly used by influencers and other content creators, whose images participants may view on social media. The selfie-stick allows for the unique selfie angle but with an increased distance from the body. Therefore, the next experiment will compare aesthetic judgements of selfies and images taken with a selfie-stick. This will allow us to compare selfie images of the body taken from close to the body to selfie images taken at further distances from the body.”*

**The used material is non-standardized. Past research could show that even small variations of angle might affect the perceived body weight.**

This comment highlighted an important limitation in the current set of experiments that had not sufficiently been considered in the discussion of the original manuscript. The main aim when developing these images was ecological validity, but that came at the cost of control. We have added a section in the discussion of the revised version that considers this limitation of our work (page 45/46).

*“Another limitation relates to the lack of standardisation in the stimuli. Photos were taken a set distance away from the model (for allocentric and egocentric images), an arm’s length away from the model (selfie) or the length of a selfie-stick away from the model. This was done to try to create ecologically valid stimuli, by producing photos that were similar to those that participants may typically see on social media platforms like Instagram. To control these parameters as much as possible, we set a standard distance from which the allocentric image was taken, set a specific level of the shoulders from which the egocentric picture was taken, set the selfie as taken from the model’s arm length away, and set a standard length of selfie-stick from which the selfie-stick images were taken. We focused on developing stimuli that were as close as possible to typical Instagram images, however, the precise angle or distance from which the photos were taken may have influenced results. Furthermore, the images were optimized for the screen they were viewed upon; the way these would have been seen on a smartphone would have differed to how they would have been viewed if participants used a desktop computer or a laptop. This may have influenced results as not only would the images be a different size, but they may also have been viewed at different brightness and other relevant screen settings. There is no available research regarding whether these factors influence our aesthetic judgements of images, so it is difficult to ascertain how this may have affected results.”*

**How did the authors control that every picture was taken from exact the same angle? If the authors belief that this is not necessary, please convince us!**

The stimuli used in this set of experiments were intended to closely mirror content that can be seen on popular social media platforms like Instagram. As a result of this, we did not control that every picture was taken from precisely the same angle, rather we focused on ensuring consistency in the perspectives that were included. We added clarification on this in the limitations section of the discussion (page 45/46)

*“To control these parameters as much as possible, we set a standard distance from which the allocentric image was taken, set a specific level of the shoulders from which the egocentric picture was taken, set the selfie as taken from the model’s arm length away, and set a standard length of selfie-stick from which the selfie-stick images were taken.”*

**6. Why did only female participants take place in this study (page 20)? Potential cross-gender differences are not discussed.**

Many thanks for highlighting this important omission that was also related to the first point concerning the title. We have added a clarifying sentence at the end of the introduction around recruiting a female sample (page 8).

*“Given that much of the research into body satisfaction, use of social media, and attractiveness judgements has used predominantly female samples, we focused on women in this study.”*

We have also now discussed this in greater detail in the discussion and amended the title to reflect an only female sample (page 46).

***“****That only female participants took part in this study could also limit our findings somewhat. We focused on women’s aesthetic judgements as all of the models for the stimuli were women and we were interested in potential impact on social comparisons and body satisfaction. It may be that ratings of these stimuli from non-female participants would differ. Future research should consider whether there is an impact of model and/or participant gender on aesthetic judgements.”*

**7. I would strongly recommend reconsidering and revising the structure of the MS. I found it puzzling in some way and therefore very hard to follow all procedures, methods, and reported result My suggestion is to split everything off into EXP 1, EXP2 etc. Regarding the results one solution could be to revise the main figures (which were lacking from important information). For a better readability the authors could add some information like effect sizes or/an p-values. This might reduce very detailed data reporting in the plain text.**

We have revised the structure of the manuscript based on this comment, which helpfully highlighted some changes we could make in order to improve the readability of the study. To do this we have written a general introduction, and then included methods, results and a specific discussion for each experiment in turn. We have then included a general discussion at the end of the manuscript. We hope that this change to the structure of the paper will make it easier to follow the procedures used as well as the hypotheses and findings from the experiments.

**8. From here the authors are consequently talking about their hypothesis. I couldn’t find a list of all hypotheses. It is obvious that some of these hypotheses are derived from the author’s theoretical framework. But this remains unclear.**

Many thanks for highlighting that the hypotheses of the study were unclear. To rectify this we have added a paragraph at the end of the introduction that clearly states each hypothesis and links these back to previous research (pages 7/8).

*“This study aimed to consider how people judge social media style content of bodies captured from different visual perspectives in a series of four separate experiments. Previous research found that selfies of faces were judged as more attractive (Noyes & Jenkins, 2017; Bryan, Perona & Adolphs, 2012) and that egocentric images of large bodies were judged as more attractive and slimmer (Carely et al. 2019). Based on this research it is hypothesised that selfies will be deemed more attractive and slimmer than both allocentric and egocentric images of the same bodies. We include four different visual angles of the body in an attempt to adequately capture the different kinds of images that are available on social media. Typically, social media platforms include photos taken by another person in an allocentric view, photos taken of one’s own body from an individual’s own perspective (an egocentric view), and selfies taken either at arm’s length or with a selfie-stick. Secondly, based on previous research indicating that participants judge others’ bodies as thinner if they have an eating disorder (Alleva et al., 2013), and that social media particularly affects those vulnerable to disordered eating (Ferguson, 2013; Aparicio-Martinez et al., 2019) it was hypothesised that aesthetic and weight judgements of social media style body images (particularly selfies) will be related to participants’ disordered eating symptomatology (Eating Disorder Examination Questionnaire (EDE-Q) score), such that aesthetically favourable judgements for selfies (attractiveness and slimness) would be stronger for those participants with greater ED vulnerability. Finally, based on previous findings suggesting that WHR and BMI may be important cues in judging attractiveness in women, and given that assumed WHR may change based on the perspective a photo is taken from (unlike BMI), it was hypothesised that differences in WHR between the perspectives will relate to differences in attractiveness judgements across perspectives. Previous research indicates that the optimal WHR is close to 0.7. Therefore, we anticipate that visual changes in WHR from changes in perspective that are closer to 0.7 will be associated with increased attractiveness judgements. Given that much of the research into body satisfaction, use of social media, and attractiveness judgements has used predominantly female samples, we focused on women in this study.”*

Using the new format, we have also more easily highlighted hypotheses specific to each experiment: page 19

*“In the current study, we therefore compared weight and attractiveness ratings for selfies and egocentric images. Due to the range of body sizes used in the current stimuli (as opposed to overweight bodies only), we do not anticipate occlusion of weight related visual cues to have a significant impact on weight and attractiveness ratings, instead we predict that selfies will be judged as more attractive compared to egocentric images of the same bodies. This is partly due to negative self-bias (Dijkstra & Barelds, 2011) and partly due to a positive bias for selfie images.”*

Page 26:

*“Therefore, the next experiment will compare aesthetic judgements of selfies and images taken with a selfie-stick. This will allow us to compare selfie images of the body taken from close to the body to selfie images taken at further distances from the body. If distance influences perception of the body in the same way as has been recorded for faces, we predict that selfies taken with a selfie stick would be judged as slimmer and more attractive.”*

Page 32

*“…the final experiment in this series compares aesthetic judgements across all four perspectives in one session predicting to replicate the above described findings”*

**9. I find it interesting that the authors revealed that the selfie condition yielded lower body weight estimations compared to the “selfie-stick” condition (shown in Fig 3). However, explanation is missing.**

The issue with the selfie vs. selfie-stick results is that different effects were found in experiment three compared to experiment four. To directly address this comment we have included a suggested interpretation for this specific effect on page 38

*“Those who use selfie-sticks are judged as less socially attractive than traditional selfie takers (Bevan, 2017). This more negative association with selfie-sticks may influence size ratings as research indicates that social attractiveness is associated with slimness (Roddy et al., 2011). This may suggest a more complex combination of factors influencing our judgments of selfie-stick compared to selfie images that could be influenced by participant individual differences and thus explain different patterns of results using different population samples.”*

However, the main interpretation surrounding selfie vs selfie-stick comparisons focuses on reasons for lack of replication:

Page 38:

*“Interestingly, these results also demonstrate that differences in weight and attractiveness judgments between selfies taken with and without selfie-sticks are less robust. This makes sense given that the angles of these two types of images are very similar compared to differences in appearance of the body between selfies and the other comparison perspectives. Moreover, even if some aspects of the of the body in selfie-stick image may appear marginally more optimal, social characteristics associated specifically with this tool may counteract this, at least for some individuals.”*

Page 41:

*“These competing observations may explain why experiment three only found increased attractiveness and not slimness for selfie-sticks compared to regular selfies; the body may appear rounder and less slim, but the social attributes linked to the appearance and position may make the image more attractive. However, those seen using selfie-sticks have also been judged as less attractive than individuals taking selfies without selfie-sticks (Bevan, 2017) and increased attractiveness for selfie-sticks was not replicated in experiment four, instead regular selfies were judged to be slimmer with no difference in attractiveness ratings. Therefore, many potential competing factors influencing how images taken with selfie-sticks are judged, alongside the fact that this is a type of image which has very little research focus, may suggest that differences between selfies and selfie-stick images are not robust and may depend on individual characteristics of the person depicted in the image as well as the person rating the image.”*

**Even if I appreciate that the authors did consider power analyses, please stay consequent and report on the verification of ALL ANOVA assumptions to convince us you can use it (e.g., independence of observations, normality of distribution of residuals as well as the homoscedasticity across and within all groups).**

In the revised manuscript we have added more details concerning the assumptions for one-way repeated measures ANOVA in the Experiment Four Results section. We have done this separately for each ANOVA.

Page 34

*“To examine difference in weight judgments across perspective we conducted a repeated measures one-way ANOVA, with a single factor of perspective (allocentric, egocentric, selfie, selfie-stick). Data met the assumption of being normally distributed according to examination of histograms and Shapiro-Wilk test (all p values > .050). Mauchly’s test indicated that the data violated the assumption of sphericity (p = .043) thus the Greenhouse-Geisser correction was used.”*

Page 35/36:

*“To examine the effect of perspective on attractiveness ratings we conducted a repeated measures one-way ANOVA on attractiveness VAS scores, with the single factor perspective (allocentric, egocentric, selfie, selfie-stick). Data met the assumption for, normality (Shapiro-Wilk p-values all > .050) and did not violate the assumption of sphericity (p-values > .050).”*

**Data reporting is not consistent across the MS and is not in line with the common APA 6th rules (e.g., SD, M or sometimes p is not italic)**

Many thanks for pointing this out! In the revised manuscript we have amended data reporting so that it is consistent and in line with APA 6th guidlines.

**Please provide line numbers! As a reviewer it is impossible to be efficient without line numbers.**

Line numbers have been added throughout the revised manuscript.

**A key reference is missing regarding weight perception and viewing angle: Schneider & Carbon (2012).**

We have added this reference to the manuscript.

**Reviewer Two Comments**

**In my opinion, the abstract might be more concise: e.g., the first sentence is not really needed, and I find the third period (“However, the influence…”) confusing, possibly too long; also, it is not immediately clear what the images perspective are supposed to influence (I imagine the authors refer to attractiveness and weight, as reported afterwards), it may be of help to the reader reporting this more explicitly.**

Based on these comments we have rewritten the abstract to improve how concise it is and make it clearer and easier to read.

*“Taking and posting selfies is a popular activity, with some individuals taking and sharing multiple selfies each day. The influence of the selfie angle, as opposed to more traditional photo angles such as the allocentric images we see in print media, on our aesthetic judgements of images of bodies has not been explored. This study compared the attractiveness and weight judgements that participants made of images of the same bodies taken from different visual angles over a series of four experiments (total N = 272). We considered how these judgements may relate to disordered eating thoughts and behaviours. Selfies were judged to be slimmer than images from other perspectives, and egocentric images were judged to be the least attractive. The way participants rated bodies seen from different perspectives was related to their own disordered eating thoughts and behaviours. These results contribute to our understanding of how we perceive the images we see on social media and how these might be related to how we feel about our own and other people’s bodies.”*

**I found quite difficult to get key information in the Introduction. Personally, I would benefit from reporting more concise and less redundant information, thus, shortening the overall length of this section.**

Many thanks for the helpful comments around the introduction and the need for it to be more concise. We have edited the introduction in the revised manuscript based on the reviewer’s comments in order to make it easier to read with less superfluous information. We have also reworked the section of the introduction that looks at evidence around judgements of faces in lines with both reviewers’ comments. We hope that this is now more clearly linked to the focus of the paper.

**“Recent research indicates that viewing more selfies is linked to facial dissatisfaction, a relationship mediated by appearance comparisons (Yang, Fardouly, Wang & Shi, 2020)” (page 12). Could the authors clarify what does it mean “mediated by appearance comparisons”?**

Based on the reviewers comments we have amended this section so it is clearer what is meant by “mediated by appearance comparisons”. Page 6:

*“Recent research indicates that viewing more selfies is linked to facial dissatisfaction, a relationship mediated by appearance comparisons such that selfies are associated with facial dissatisfaction through increased appearance comparisons towards these images (Yang, Fardouly, Wang & Shi, 2020).”*

**3. Page 12, on the meta-analysis by Ferguson (2013): I apologize, but it is hard to understand the point made about effect size, could the authors clarify this point and clearly link this digression to their own study? Also, beyond the issue concerning effect size, it may be noted that the metanalysis mentioned is quite dated and, conceivably, the studies included are even older; however, the use of social media changed rapidly. I strongly encourage the authors to provide more recent evidence on this issue (e.g., at a first look on pub med the following might be of interest: 10.3390/ijerph20043484 and 10.3390/ijerph16214177). This may give stronger support to the authors’ hypothesis of a correlation between EDs and social-media images ratings (also, please note that eating disorders were initially abbreviated in EDs and then ED was used)**

The above comment was very helpful in thinking about the structure of the introduction, in particular considering social media and eating disorders. Based on reviewer feedback we have edited this section and included more recent references that point to the relationship between use of social media and disordered eating. Upon reflection, we have also removed the section around effect size and moved this consideration of effect size to the discussion of the studies instead. (now page 8)

*“based on previous research indicating that participants judge others’ bodies as thinner if they have an eating disorder (Alleva et al., 2013), and that social media particularly affects those vulnerable to disordered eating (Ferguson, 2013; Aparicio-Martinez et al., 2019) it was hypothesised that aesthetic and weight judgements of social media style body images (particularly selfies) will be related to participants’ disordered eating symptomatology (Eating Disorder Examination Questionnaire (EDE-Q) score), such that aesthetically favourable judgements for selfies (attractiveness and slimness) would be stronger for those participants with greater ED vulnerability.”*

**Also, I found information in support of this hypothesis a bit scattered across the introduction. I think the purposes of the study may be clearer focusing first on the perspective-related evidence, which it seems to me the main aim of the study and how the authors addressed this research question; then, focusing on the evidence in support of the hypothesis of a possible ED/ratings link, thus introducing the secondary aim of the study and how it was probed.**

Based on this feedback we have completely re-worked the structure of the introduction so that perspective-related evidence is addressed first, followed by a brief outline of the evidence in support of the ED/ratings link..

**6. Finally, I was surprised by the fact that women with more optimal WHR ratios were judged as less attractive, I wonder whether the authors have a cue about that (just curiosity!**

We apologies for this lack of clarity. The results are actually in the hypothesized direction (attractiveness linked to a more optimal WHR of 0.7). We have clarified this now at several points within the MS including the introduction Page 8:

*“Previous research indicates that the optimal WHR is close to 0.7 (Streeter & McBurney, 2003). Therefore, we anticipate that visual changes in WHR from changes in perspective that are closer to 0.7 will be associated with increased attractiveness judgements.”*

The data analysis section for experiment one Page 15:

*“Because the majority of the bodies in our stimuli had a WHR > .07 and we anticipate that selfies would be associated with more optimal WHR, negative correlations between attractiveness and WHR differences would represent relationships in the predicted direction (greater attractiveness would be associated with WHR’s closer to optimal in selfie images).”*

The results section of experiment two (where we found a significant correlation). Page 23

*“… such that more optimal WHR was related to higher attractiveness judgements for selfie images compared to for egocentric images. Given the ranges of WHR for these conditions, this relationship represents increased attractiveness being associated with more optimal WHR measured in the selfie images.”*

The data analysis section for experiment three (requiring a slightly different calculation) Page 28.

*“For the WHR we subtracted WHR for selfie images from the WHR from selfie-stick images. Therefore, negative correlations represented relationships in the direction of our hypothesis (greater attractiveness being associated with more optimal WHR for selfie-stick images).”*

And in the results section for experiment three (where we also found a significant correlation. Page 30.

*“…such that more optimal WHR was related to higher attractiveness judgements for selfie-stick images compared to for selfies.”*

**Overall, I found the organization of this section quite confusing.**
**7. Reporting power analysis before stating the procedure and the analyses performed seems awkward since this are key elements for establishing the sample size.**
**8. Also, In the Participants section I expected to see the overall (i.e., common to the four experiments) inclusion/exclusion criteria and the information on recruitment modalities, which are report later on (e.g., some information is reported in the Procedure). I suggest reporting here the information which are common to the four experiments and then add study-specific information when talking about each of the experiment. Moreover, I think it may be more logical to report the recruitment procedure in the Methods and then results about the sample in the Results section (after the sample size calculation). I understand the authors reported here the power analysis to introduce the sample; however, in my opinion, the structure I suggest may improve readability, please consider this format.**

Based on both reviewers’ comments around the structure of the methods section and indeed of the full manuscript, we have reshaped this section. In line with the helpful comments received we have moved power analysis for experiments 1-3 (page 9) and experiment 4 (page 32) to a more relevant section. We have also included the overall information around inclusion/exclusion criteria and information on recruitment to the beginning of the methods section page 9:

*“All four experiments had the same inclusion criteria (identifying as female, being 18 and over) and exclusion criteria (history of an eating disorder, being under 18).”*

**9. Please, clarified whether only females were recruited.**

We have made it clearer in the revised manuscript that only women were recruited for each experiment.

Including a statement in the introduction Page 8:

*“Given that much of the research into body satisfaction, use of social media, and attractiveness judgements has used predominantly female samples, we focused on women in this study.”*

As well as addressing this as a limitation in the discussion Page 46

*“That only female participants took part in this study could also limit our findings somewhat. We focused on women’s aesthetic judgements as all of the models for the stimuli were women and we were interested in potential impact on social comparisons and body satisfaction. It may be that ratings of these stimuli from non-female participants would differ. Future research should consider whether there is an impact of model and/or participant gender on aesthetic judgements.”*

And in response to a similar point from another reviewer we have also change the title to reflect this

“*Do selfies make women look slimmer? the effect of viewing angle on aesthetic and weight judgments of women’s bodies.* ”

**10. Page 16 (Materials): when describing the EDE-Q authors mentioned four subscales (Restraint, Eating Concern, Shape Concern, and Weight Concern). However, when reporting the Results it seem that Shape and Weight Concerns were merged. I could not find any mention/explanations of this. Could the authors clarify this point? Also, could the authors make explicit the reason why the six “additional” items were not taken into consideration in this study?**

Many thanks for helpfully pointing out these points. In the revised manuscript we have made it clearer that we are using a three factor model of the EDE-Q, alongside clarifying why the six descriptive items were not included. Page 10

*“However, more recent research indicates that a three-factor model is a better fit for the data, especially in non-clinical samples (Carey et al., 2019; Knight et al., 2022). Based on this, we use a three-factor model that combines the two Shape Concern and Weight Concern factors into one. Participants rate items on a 7-point Likert scale, with higher scores indicating higher eating disorder psychopathology. There are six items that relate to the frequency of eating disorder attitudes and behaviours in the past 28 days, which do not contribute to the subscale or global scores but provide information on some core eating disorder behaviours such as laxative use and self-induced vomiting. These are not used in this study. This is partly for ethical reasons, as the responses to these questions do not contribute to the subscale scores, and partly as the information collected often provided more qualitative responses to the questions.”*

**11. Page 18: the authors reported that “age, gender, and nationality were asked: were male participants excluded? Also, was nationality an exclusion criterion? Finally, were the height/weight measurements asked after the task because the author believe asking for one’s own weight before the task possibly affect ratings?**

Based on these useful comments here and above we have clarified the inclusion and exclusion criteria Page 9

*“All four experiments had the same inclusion criteria (identifying as female, being 18 and over) and exclusion criteria (history of an eating disorder, being under 18).”*

As well as explaining why height and weight were reported at the end of the experiment. Page 13

*“Participants then completed the EDE-Q and recorded their own self-reported weight and height (to calculate BMI)”*

**12. Page 18: Please, provide more details about the procedure. For example, when stating that “the order of these blocks was randomized” do the authors mean the judgment required (attractiveness vs weight) was randomized across participants, thus images were judged concerning attractiveness first and weight after that (and vice versa) whereas “perspectives” was randomized within each block? Were judgments (attractiveness vs weight) repeated four time in each participant for each image? This was true also in experiment four (it is not clear whether the block was repeated four times)? How many trials were included overall? There were constraints or a specific timing relative to the images presentation? Could the authors provide information about the features of the images (e.g., size?). I imagine the size/format of the images depend on the device used: do the authors control for this factor? If not, I suggest discussing the possible implication of this point among the study limitations. For instance, viewing an image on a 15” laptop may be different than viewing them on a smartphone, do the authors believe that this possibly affect ratings? Overall, I think this section may be clearer reporting the information common to the four experiments only once and then focusing on differences (e.g., kind of pictures included).**

We have revised the methods section of the manuscript in line with the reviewers comments here. As well as adapting the procedure sub-section to report common characteristics and then specifically reporting any differences by experiment, we have also added more information on the procedure of the study. Alongside this, we have clarified in the methods section that images were optimized for the screen participants viewed them on (underlined statement below). Page 13

*“Participants accessed the experiments via a personalised link through Qualtrics (Qualtrics, Provo, UT) that was sent to their university email address. When following the link, participants first answered demographic questions regarding age, gender, and nationality, followed by instructions for the experimental task. Within each block, they were presented with 10 images of bodies from the same visual perspective in succession and for each image were asked to rate the attractiveness and weight of these bodies on a visual analogue scale (VAS) ranging from 0 to 100 (in separate blocks). The VAS were anchored with ‘Very Unattractive’ and ‘Very Attractive’ for the attractiveness ratings, and ‘Very Underweight’ and ‘Very Overweight’ for the weight ratings. Participants used their mouse to select the position on the scale that they felt best represented the attractiveness or weight of the body in the photo that was displayed. The image was present on the screen for as long as the participant took to make the judgements. After the judgement was made, the next photo and VAS were displayed. All images were optimized to fit the screen that the participant was viewing them on (participants could access the questionnaire using their smartphone).*

*Participants judged weight and attractiveness for two perspectives in this experiment; selfie and allocentric images, thus the experiment consisted of four separate experimental blocks: selfie weight judgments, selfie attractiveness judgments, allocentric weight judgements and allocentric attractiveness judgments. The order of these blocks was randomised and images within the blocks were also presented in a random order. Participants then completed the EDE-Q and recorded their own self-reported weight and height (to calculate BMI) before finishing the questionnaire and being debriefed. Weight and height were completed at the end along with the EDE-Q to mitigate any potential effect of answering these body focussed questions on aesthetic judgements during the experiment.”*

In line with the reviewer’s useful comment around the way this may affect judgements, we have added a short section on this point when considering limitations in the discussion section of the manuscript. Page 46

*“Furthermore, the images were optimized for the screen they were viewed upon; the way these would have been seen on a smartphone would have differed to how they would have been viewed if participants used a desktop computer or a laptop. This may have influenced results as not only would the images be a different size, but they may also have been viewed at different brightness and other relevant screen settings. There is no available research regarding whether these factors influence our aesthetic judgements of images, so it is difficult to ascertain how this may have affected results.”*

**13. Also, please note that the name of EDE-Q is reported inconsistently across the manuscript (sometimes reporting the version 6.0 and others not)**

We have edited the manuscript to ensure the EDE-Q is named consistently throughout.

**14. Finally, please clarify whether in experiment four the EDE-Q was not collected, was it because the participants were those (i.e., some of) included in the other experiments?**

We have clarified that the EDE-Q was not collected in experiment four in the methods Page 33

“Due to time constraints, the EDE-Q was not included in this experiment.”

and also more clearly referred to this in discussion of the experiment’s limitations. Page 38

*“As EDE-Q responses were not collected in this experiment due to time constraints, it is not possible to ascertain the links between these difference in aesthetic judgements and disordered eating thoughts and behaviours.”*

And on page 44/45

*“There are several limitations to this series of experiments. Initially two perspectives were compared with each other in three separate experiments, as well as collecting EDE-Q scores. In the final experiment however, participants saw images from all four perspectives, but did not fill in the EDE-Q. It would have potentially been more effective to recruit a larger number of participants who saw all stimuli and also completed the EDE-Q so we could attempt to replicate not only the effects of perspective, but also the correlational relationships with eating disorder thoughts and behaviours.”*

**15. Analysis: I strongly encourage the authors in making separated paragraphs concerning experiments one to three and the fourth since analyses were different and it is not clear what was done in which experiment (e.g., in the first sentence authors mentioned EDE-Q subscales – but this was done only in experiments one to three). Furthermore, I would find easier reporting/discussing the “topics” always in the same order across all the sections. For example, starting always with perspective comparisons (in the introduction, then methods, results and discussion) and then introducing the EDE-Q part. This may also help the reader to differentiate more clearly between primary and secondary aims (if there is a hierarchy) and/or follow the contents while reading. This may be true even tough – temporally speaking - the authors computed before the EDE-Q scores and the average ratings. So that, starting with overall consideration about the analyses (e.g., which software was used etc), then presenting the analyses concerning the experimental protocols, separately for experiment one-three and the fourth and finally everything concerning the EDE-Q scores and correlations.**

Based on these helpful comments, alongside comments from the first reviewer, we have restructured the manuscript so that the methods, results, and discussion of each experiment are outlined separately. Furthermore, we have followed the reviewers’ suggestions to discuss the topics in the same order for each experiment to improve clarity and readability.

**16. Concerning correlation, authors should clarify what they mean with “differences” in judgments. I would suggest reporting here (rather than later on) how, and why, the authors computed this additional variable. As I understand this is always a difference in attractiveness and weight between the two perspectives adopted; therefore, the authors may state this computation here in the analyses section (when illustrating experiment one to three) and then in each experiment provide only the relative interpretation of the delta (e.g., positive values means….(i.e., perspective 1- perspective 2) etc. to reduce redundant information.**

Based on these comments we have clarified what is meant in terms of differences between judgements (ie these refer to differences in attractiveness judgements across perspectives and differences in weight judgements across perspectives). We have clarified how these differences were computed in the data analysis sections of the manuscript and only provided the interpretations along with the correlation information in the results sections. We continued to keep individual descriptions of this calculation for each experiment, due to experiment three hypothesising slimmer and more attractive judgments in a favour of selfie-sticks rather than traditions selfies so thought that this reduced potential confusion.

Experiment one Page 14

*“Attractiveness difference scores were calculated by subtracting attractiveness scores for allocentric images from selfie images such that positive scores represented greater attractiveness judgements for selfie images and negative scores represented greater attractiveness for allocentric images. The reverse calculation was done for the weight rating such that positive scores represent slimmer judgements for selfies and negative scores represent slimmer scores for allocentric images.”*

Experiment two Page 21/22

*“Attractiveness differences were calculated by subtracting scores for egocentric images from selfie images such that positive scores represented greater attractiveness judgments for selfie images and negative scores represented greater attractiveness judgements for egocentric images. The reverse calculation was done for the weight ratings such that positive scores represent slimmer judgements for selfies and negative scores represent slimmer scores for egocentric images”*

Experiment three page 28

*“attractiveness difference scores were calculated by subtracting scores for selfie images from selfie-stick images such that positive scores represented greater attractiveness ratings for selfie-stick images and negative scores represented greater attractiveness ratings for selfie images. The reverse calculation was done for the weight rating such that positive scores represent slimmer judgements for selfie-stick images and negative scores represent slimmer scores for selfie images.”*

Where significant correlations were detected we also included clarification in the results.

Experiment one, page 17

*“…there were significant correlations between attractiveness differences and Shape and Weight Concern scores (τb = 0.21, p = .011) and between attractiveness differences and Preoccupation and Eating Concern scores (τb = 0.23, p = .009), such that greater SWC and PEC scores were related to greater attractiveness ratings for selfies compared to allocentric images.”*

Experiment three, page 29/30

*“..there was a significant correlation between attractiveness differences and Restriction scores (τb = 0.35, p = .022) such that higher Restriction scores were associated with greater attractiveness ratings for selfie-stick compared to regular selfie images.”*

**17. Page 20: when discussing data normality, please clarify the dependent variables considered (instead referring to “results” in general). Also, I would appreciate reporting Shapiro-Wilk p values (for each variable) or at least an overall p value (e.g., all p values < [the highest one]); this may give raw information about the significance of deviation**

We have amended the results sections in the revised manuscript to make it clearer which variables are considered, as well as giving an overall Shapiro-Wilk p value. This has been done for each results section, and the section from experiment one is included below as an example.

Experiment one, Page 16

***“****Data for all variables (attractiveness judgements, weight judgements, differences in attractiveness judgements across perspectives, differences in weight judgements across perspectives, and EDE-Q scores) were not normally distributed, according to Shapiro-Wilk tests (largest p value: p = .021) and analysis of histograms. Because of this, Wilcoxon signed-rank tests were used.”*

Experiment two, page 22

*“According to Shapiro-Wilk tests and examination of histograms, selfie weight averages, selfie attractiveness averages, egocentric attractiveness averages, Restriction subscale scores, Shape and Weight Concern subscale scores, and Preoccupation and Eating Concern subscale scores were not normally distributed (greatest p value: p = .045).”*

Experiment three, page 28

***“****Shapiro-Wilk tests and examination of histograms showed that selfie weight average ratings, selfie attractiveness average ratings, weight rating differences, Restriction scores, and Preoccupation and Eating Concern scores were not normally distributed (largest p value: p = .023).”*

**18. Page 20: as mentioned, I suggest moving information about the EDE-Q subscale validity when discussing the results of this scale. It may be useful also to report references for the validity cut-off adopted.**

Based on these comments we have moved information about EDE-Q subscale validity,along with references for validity cut-offs, to the part of the results section that focuses on results of the scale.

Experiment one, page 16

*“The EDE-Q subscales demonstrated good internal consistency in this sample using both Mcdonald’s ω and Cronbach’s alpha (Shape and Weight Concern ω = .939, α = .937; Preoccupation and Eating Concern ω = .714, α = .708; Restraint ω = .0.764, α = .0.735) (Hayes & Coutts, 2020; Ravinder & Saraswathi, 2020).”*

Experiment two, page 22

*“The EDE-Q subscales demonstrated good internal consistency in this sample using both Mcdonald’s ω and Cronbach’s alpha (Shape Concern and Weight Concern ω = .958, α = .956; Eating Concern ω = .884, α = .875; Restraint ω = .0.886, α = .0.875)”*

Experiment three, page 29

*“The EDE-Q subscales demonstrated good internal consistency in this sample using both Mcdonald’s ω and Cronbach’s alpha (Shape Concern and Weight Concern ω = .958, α = .956; Eating Concern ω = .884, α = .875; Restraint ω = .0.886, α = .0.875)”*

**19. When reporting the experiments 1-4 results, it would be advisable to show means also of non-significant effects. If the authors believe this may reduce readability, they may provide all the means in a table (specifying whether rank means are displayed when non-parametric tests are used). In this regards, standard deviations may be always reported.**

Many thanks for this helpful comment. We have added means and standard deviations of non-significant effects, and have also ensured that standard deviations are consistently reported alongside means.

**Results about WHR correlations are confused: it is not clear which variables were correlated, differences in WHR were computed between the two perspective? This should be clearly reported in the analysis. Also, data on WHR (beyond correlational statistic) should be reported.**

We have clarified in the data analysis sections which variables we tested to see if they were correlated, and how these were calculated.

Experiment one (this is also referred to rather than being repeated for experiment two) page 14/15

*“To do this we subtracted WHR for allocentric images from selfie image and correlated these with the attractiveness differences as calculated above. Because the majority of the bodies in our stimuli had a WHR > .07 and we anticipate that selfies would be associated with more optimal WHR, negative correlations between attractiveness and WHR differences would represent relationships in the predicted direction (greater attractiveness would be associated with WHR’s closer to optimal in selfie images).”*

Experiment three page 28

*“For the WHR we subtracted WHR for selfie images from the WHR from selfie-stick images. Therefore, negative correlations represented relationships in the direction of our hypothesis (greater attractiveness being associated with more optimal WHR for selfie-stick images).”*

We also added data on the WHR to the report, including means and standard deviations for further clarification.

**21. I strongly suggest keeping the same order of presentation of results for all experiments; also, it would be helpful referring explicitly to the Table 1 (please define BMI units (i.e., kg/m2) here) when describing the sample of each experiment (As previously mentioned, I believe samples should be described for each experiment independently, before showing the experimental results).**

In line with these helpful comments and those from the other reviewer we have restructured the manuscript, including standardising the order of presentation for the results of all experiments. In the revised manuscript, the results from each experiment are addressed individually, and all results sections consider participant information, normality tests, comparing weight and attractiveness judgements across perspectives, correlations with EDE-Q scores, and finally correlations between attractiveness judgements and WHR in that order. We hope that this improves the readability of the results.

**22. Experiment 4: please, report more information about the assumptions check. Importantly, the bayes analyses were not introduced – nor justified - in the analysis section. Also, please give interpretation of bayes factor with proper reference (since it may not be acknowledge by all readers). Finally, since the BF was computed, it may be worthy commenting this briefly in the discussion.**

**I strongly suggest the authors to apply my previous considerations about organization also to this section. Since there are many experiments it may be of help discussing them once a time and then make overall considerations (a possibility is also to provide single a discussion for each experiment and then a “general discussion”, this may guide the reader along the results). Beyond this, I suggest the following structure: initial hypotheses > results > discussion/interpretation relative to hypotheses and previous literature; specifically, this may be done for experimental data, EDE-Q and WHR correlations following the same order adopted in the previous sections.**

We have included information regarding ANOVA assumptions for both attractiveness and weight analysis

Page 34

*“To examine difference in weight judgments across perspective we conducted a repeated measures one-way ANOVA, with a single factor of perspective (allocentric, egocentric, selfie, selfie-stick). Data met the assumption of being normally distributed according to examination of histograms and Shapiro-Wilk test (all p values > .050). Mauchly’s test indicated that the data violated the assumption of sphericity (p = .043) thus the Greenhouse-Geisser correction was used.”*

Page 35/36:

*“To examine the effect of perspective on attractiveness ratings we conducted a repeated measures one-way ANOVA on attractiveness VAS scores, with the single factor perspective (allocentric, egocentric, selfie, selfie-stick). Data met the assumption for, normality (Shapiro-Wilk p-values all > .050) and did not violate the assumption of sphericity (p-values > .050).”*

We have also introduced the BF

Page 33/34

*“We calculated Bayes factors (BF) of any non-significant results to determine the likelihood of the data occurring under the null hypothesis (Kass & Raftery, 1995). The BF represents a likelihood ratio of the alternative relative to the null hypothesis (*[*Dienes, 2014*](https://www.sciencedirect.com/science/article/pii/S0022096522001060#b0085)*). A BF > 3 indicates evidence for the alternative hypothesis, a BF < 0.33 indicates evidence for the null hypothesis and a BF between 0.33 and 3 being inconclusive (*[*Dienes, 2014*](https://www.sciencedirect.com/science/article/pii/S0022096522001060#b0085)*).”*

As well as refering to this analysis on the discussion page 38

*“BF were inconclusive for the non-significant comparisons, although this does not provide statistical support for the null hypothesis, these results also do not support the alternative hypotheses, such that any effect of perspective in these comparisons is likely to be very small.”*

We have also revised the MS as suggested

**26. Page 28: “Results suggested that there are clear differences between selfies and egocentric images, with the latter judged as both slimmer and more attractive.” It seems to me that these results are not coherent with those reported previously, could the authors check this point?**

Many thanks for pointing out our error, this has been removed from the manuscript.

**27. Also, I believe that the results might be discussed more deeply, relative to hypothesis (why there are/are not confirmed? What does the literature say?)**

Based on this feedback we have amended the manuscript so that the discussion considers the results in greater depth. We have made these changes within the separate discussions for each experiment as well as in the general discussion.

**28. Page 31: as previously pointed out, I found discussing evidence concerning the face, without focusing on that in the study were used body images (i.e., without comparing face-related evidence with the study results), quite misleading. Could the authors enhance the link between face-related evidence and their results?**

Many thanks for underlining that the links between face and body perception needed to be made clearer so as not to be misleading. We have clarified the link between the two and revised the manuscript so that the conclusions are not predicated on findings from face perception research. Page 41

*“In experiment three, selfie-stick images were judged to be more attractive than typical selfie images. This may be related to the photo being taken from further away. Research into face perception has shown that the distance the face is from the camera affects attractiveness judgements of the face, with faces seen from further away being deemed more attractive than those seen close up (Noyes & Jenkins, 2017; Bryan, Perona & Adolphs, 2012). Therefore, it may be that distance from the camera could influence attractiveness judgements of bodies in a similar way. Due to selfie-sticks placing the subject's body further away from the camera compared to traditional this may explain more attractive judgments for selfie-stick images. However, the mechanism through which increased attractiveness of the face is thought to be associated with increased distance is that when further away faces appear more convex and so may appear as rounder (Noyes & Jenkins, 2017). Whereas this may soften features of the face, if the same effect is also applied to bodies, it could make bodies appear larger and a less socially desirable (slim with flat stomach) shape.”*

**29. Page 32: “Interestingly, changes in slimness did not always have a corresponding change in attractiveness ratings for selfies” do the authors test this hypothesis, or is there evidence in literature?**

Many thanks for this useful feedback. This referred significant differences in slimness between perspective but no significant differences in attractiveness within the same experiment (our study). This has been clarified. Page 41

*“Interestingly, significant difference in slimness between perspectives did not always have a corresponding significant difference in attractiveness ratings in these experiments.”*

**30. Page 32: please when discussing correlations, please report whether they are positive or negative. Also, the authors state: “The correlations may mean that those who are more vulnerable to eating disorders might be more vulnerable to the effects of social media, such that those experiencing more thoughts and behaviours around disordered eating are more likely to judge social media style images like selfies as more attractive. I found this observation interesting and with high clinical relevance, however, I suggest rewording this sentence since in this form it seems to evoke a causal link between the two variables, which could not be probed with correlational analyses**

Based on this helpful feedback we have ensured that discussion around correlations specifically states whether these are positive or negative. We have also rewritten this passage to ensure it does not infer causality.

**31. Concluding I would appreciate if authors could further elaborate on possible clinical/social implications of their results, to enhance the relevance of their study.**

We have integrated further details around possible clinical and social implications throughout the general discussion, and have also added the paragraph to summarize these more clearly. Page 47/48

*“This research may have implications relevant to clinical practice. The relationship between social media and body satisfaction has received much research attention, but there has been less focus on the specific characteristics of social media content that may be having an effect on feelings towards the body. These results highlight that particular kinds of content may influence body satisfaction in different ways based how viewers judge the attractiveness and weight of the bodies represented in it. Those who have a pre-existing vulnerability to disordered eating may be more likely to be negatively influenced by images of bodies that they deem to be more attractive or slimmer than their own and thus that selfies may be particularly detrimental. Clinicians and educators may be able to use these results to help educate social media users around the kinds of effects different social media content may have on their body image. Specifically, our findings suggest that any preventative or educational measures applied to social media not only should consider type of content in terms of broader categories (e.g., body vs. non-body content) but also the nature of visual angle that bodies that are represented. For example, increasing awareness that the bodies we see on social media may appear slimmer than they are in reality, particularly in selfies, may help to lessen any negative impact of these images. Awareness campaigns around the high numbers of filters applied to many images found on social media have helped to give content consumers knowledge that the individuals portrayed in these images most likely do not look that way in reality. Thus, awareness of the potentially misleading effects of visual angle (selfie) may do the same, helping users to interpret the images more accurately.”*
