# Supplementary material for: Do selfies make women look slimmer? The effect of viewing angle on aesthetic and weight judgments of women’s bodies
Source: PLoS One. 2023 Oct 11;18(10):e0291987. doi: 10.1371/journal.pone.0291987 (PMC10566732; doi:10.1371/journal.pone.0291987)
Supplement: Attachment — Submitted filename: Response to Reviewers.docx [file pone.0291987.s003.docx]

**Response to Reviewers: Do selfies make women look slimmer? the effect of viewing angle on aesthetic and weight judgments of women’s bodies.**

**PONE-D-22-21961R1**

Many thanks for the helpful comments provided by the reviewer. We have addressed each of the comments below, and provided the appropriate passages from the revised manuscript in italics where suitable.

**In the description of each experiment there are elements which are common to the three of them (either repeated in each paragraph or reported only in experiment one and then referenced below). Detecting all common factors to experiment one-to-three and illustrating these shared aspects/procedures (analyses, stimuli, etc.) before describing each single experiment would further simplify the manuscript structure and reduce redundancy, improving the manuscript intelligibility. For example, the description of the EDE-Q, which is reported in experiment one methods and then referenced two times in the methods of experiment two and three, may be described only once before explaining the singularities of each experiment, clarifying that the same measure was included in experiment one-to-three.**

Based on this review we have gathered together the common aspects to each experiment in a ‘general methodology’ section at the beginning of the methods section. Then we have identified only the elements that are different in each experiment’s individual methods section (pages 15, 16, 22, 28, 29, 33, 34).

***General Methods***

*Ethics statement*

*Ethical approval for all experiments within this study was granted by the Departmental Ethics Board in the Psychology Department at the University of York. Written informed consent was given by all participants who took part.*

*Participants*

*Participants were recruited via adverts on social media and through a departmental system to allow students to participate in experiments to gain course credit. All participants who took part were students at the university. All four experiments had the same inclusion criteria (identifying as a woman, being 18 and over) and exclusion criteria (history of an eating disorder, being under 18).*

*Materials*

*Measures*

*Eating Disorder Examination Questionnaire 6.0 (EDE-Q).*

*The EDE-Q is a 28-item self-report questionnaire that assesses eating disorder symptoms in the last 28 days (Fairburn & Beglin, 1994; 2008). It traditionally uses four subscales (Restraint, Eating Concern, Shape Concern, and Weight Concern) as well as a global score, which is calculated from the mean of the four subscale scores. However, more recent research indicates that a three-factor model is a better fit for the data, especially in non-clinical samples (Carey et al., 2019; Knight et al., 2022). Based on this, we use a three-factor model that combines the two Shape Concern and Weight Concern factors into one. Participants rate items on a 7-point Likert scale, with higher scores indicating higher eating disorder psychopathology. There are six items that relate to the frequency of eating disorder attitudes and behaviours in the past 28 days, which do not contribute to the subscale or global scores but provide information on some core eating disorder behaviours such as laxative use and self-induced vomiting. These are not used in this study. This is partly for ethical reasons, as the responses to these questions do not contribute to the subscale scores, and partly as the information collected often provided detailed qualitative responses to the questions. Research has established acceptable levels of internal consistency for global and subscale scores of this three-factor model in men and women, alongside the reliability of the scale (Carey et al., 2019; Knight et al., 2022). Experiments one to three used this scale.*

*Stimuli*

*Colour photographs were taken of 10 female models’ bodies (excluding the head) standing against a white backdrop from different angles on a Samsung tablet. The models were students attending the Psychology department at the University of York and received course credit for taking part. Model BMI ranged from 18.5 to 30.6 (M = 22.46, SD = 4.23). Models were asked to wear form-fitting clothes that they would exercise in. Most models wore leggings and a form-fitting vest top. Models stood with their arms loosely by their sides with their right leg pointed slightly outwards to simulate photos commonly seen on social media. This pose was maintained for every photograph. For the allocentric angle, the experimenter stood roughly two metres in front of the model capturing the whole body. The photo for the egocentric angle was taken by the model by angling the camera down towards the body from just below the chin. The selfie-stick angle photo was again taken by the model by holding the selfie-stick an arm’s length away from the body in front of them. The model took the selfie angle with the tablet held in their left hand an arms-length away angled from above (see Figure 1). Experiments one to three used stimuli from the selfie category and one of the three other categories. Experiment four used all stimuli.*

*Procedure*

*Participants accessed all of the experiments via a personalised link through Qualtrics (Qualtrics, Provo, UT) that was sent to their university email address. When following the link, participants first answered demographic questions regarding age, gender, and nationality, followed by instructions for the experimental task. Within each block, they were presented with 10 images of bodies from the same visual perspective in succession and for each image were asked to rate the attractiveness and weight of these bodies on a visual analogue scale (VAS) ranging from 0 to 100 (in separate blocks). The VAS were anchored with ‘Very Unattractive’ and ‘Very Attractive’ for the attractiveness ratings, and ‘Very Underweight’ and ‘Very Overweight’ for the weight ratings. Participants used their mouse to select the position on the scale that they felt best represented the attractiveness or weight of the body in the photo that was displayed. The image was present on the screen for as long as the participant took to make the judgements. After the judgement was made, the next photo and VAS were displayed. All images were optimized to fit the screen that the participant was viewing them on (participants could access the questionnaire using their smartphone or computer).*

*Participants judged weight and attractiveness for two perspectives in each of the first three experiments, thus the experiment consisted of four separate experimental blocks: eg selfie weight judgments, selfie attractiveness judgments, allocentric weight judgements and allocentric attractiveness judgments. The order of these blocks was randomised and images within the blocks were also presented in a random order. Participants saw each image once, thus viewing ten images in each condition/block, 40 trials overall for experiments one to three. In experiment four, participants were shown all images across each condition and were asked to make aesthetic judgements of each one (80 trials overall.)*

*Participants then completed the EDE-Q and recorded their own self-reported weight and height (to calculate BMI) before finishing the questionnaire and debrief. BMI was calculated to compare participants to the general population (we did not compare participant and model BMI.) Weight and height were completed at the end of the study along with the EDE-Q to mitigate any potential effect of answering these body focussed questions on aesthetic judgements during the experiment, aside from for experiment four, for which the EDE-Q was not completed due to time constraints.*

**I strongly suggest the Authors to revised this sentence, which in the present wording suggests that women intentionally try to appear submissive to the purpose of attracting dating partners (indeed, the paper mentioned by the Authors specifically focused on selfies posted on Tinder). Beyond resulting absolutely disqualifying for women, this was not the interpretation given by Sedgewick and colleagues. Briefly reading their work, I understand that males and females tend to prefer poses in which they seem higher/smaller (respectively) according to (possibly covert) expectations about the height preferred by each counterpart. Then, the authors hypothesized why women may preferred height men and men might prefer smaller women (i.e., suggesting that men may prefer smaller women because they (the men) associate smaller women to faithfulness and subordination). In other words, women might try to appear smaller (consciously or not) because this is men’s preferred height, but there is no evidence - at least in the mentioned work - that women intend to seem submissive. Also, I would specify that the mentioned work only referred to pictures posted for dating purposes; in fact, the Authors may find interesting the more recent findings and considerations provided by Soranzo and Bruno (10.1371/journal.pone.0238588).**

This was a very helpful suggestion that allowed us to clarify exactly what was found in the aforementioned study. The additional reference has also been of great use in revising the manuscript, and we are grateful to the reviewer for highlight it. Based on the reviewer’s suggestions we have redrafted this paragraph to be clearer and more accurate (page 5).

*Research suggests that on dating applications such as Tinder men are more likely to use selfies taken from below, whereas women are more likely to use selfies taken from above (Sedgewick, Flath & Elias, 2017.) These results have been partly replicated and extended to include selfies in other contexts such as Instagram. When considering the context that selfies are posted in, women are more likely to post frontal selfies on Tinder compared to Instagram, and men are less likely to post selfies from below on Instagram (Soranzo & Bruno, 2020.) This seems to suggest that the context and communication intentions related to selfies influence the angle they are presented from; the authors suggest that selfies are a form of non-verbal communication in this way (Soranzo & Bruno, 2020.) On social media, for example, men and women may use selfies taken from below or above respectively due to the role of partner height in attracting potential partners (Soranzo & Bruno, 2020.)*

**I agree that most of the literature on body-related topics focused on females only; however, I believe this fact is not a solid reason per se to include only women in the sample (in fact, it may encourage more studies on men!). What I mean, is that the Authors should clarify why considering women and men separately is necessary (which is the reason why this was frequently done in the literature on body representation) and why they specifically choosed to investigate females rather than men (e.g. are they more sensitive to social medial influence? Are they more disposed to body image issues?)**

In line with the reviewer’s suggestion we have made the rationale for focusing solely on women in this study clearer (page 9).

*Research indicates that appearance pressures and patterns of body satisfaction differ between men and women, and that body ideals may be more strongly linked to other factors, such as sexual orientation, in men compared to women (Knight & Preston., 2023.) Women are typically subject to the thin-ideal, whereas for men muscularity, leanness, or both may be the dominant appearance ideal (Knight et al., 2022.) Alongside these differences in appearance pressures, there may also be differences in the way men and women present themselves on social media (Soranzo & Bruno, 2020.) Due to this, and the relatively larger amount of women who use social media (Willoughby, 2008; Pew Research Centre, 2015) we focus solely on women in this study.*

**Could the Authors clarify whether social media adverts were limited to students or none "external" participant answered the call?**

We have made it clearer that only students took part in the studies (page 9).

*Participants were recruited via adverts on social media and through a departmental system to allow students to participate in experiments to gain course credit. All participants who took part were students at the university.*

**I renew my suggestion of providing sample size analyses after the explanation of the analyses planned.**

We have provided sample size analyses after the explanation of planned data analysis as recommended by the reviewer. The extract below is taken from the general data analysis section.

*A power analysis was conducted using R studio (package pwr) (RStudio, 2020) based on detecting a medium effect size in paired samples t-tests (as shown in previous studies comparing attractiveness and weight ratings (Carey et al., 2019)). This suggested that the first three experiments would need at least 33 participants (power = 0.80, alpha of .050, d = 0.50).*

**Also, I suggest the Authors clarifying why they included more participants than the number identified. Sticking to a priori sample size calculation is a good research practice to avoid arguable "results seeking" by adding participants progressively. I am not insinuating at all that this was what the Authors did, but by clarifying the reason behind this choice may safeguard themselves.**

We thank the reviewer for this useful question. We recruited more participants than the power analysis suggested, to allow for the possibility of detecting smaller effects, which we expected we might find given the complex nature of disordered eating and social media behaviours. We also wanted to ensure that we recruited sufficient participants to allow us to discard any responses that did not fully complete the experiment, as this is a common occurrence across many online studies. Ultimately, though, all participants completed the whole experiment, which may be related to recruitment through the departmental course credit system and our experimental design ensuring that participants were not overburdened. We have added mention of this in both the data analysis section and the discussion. The below passage is taken from the data analysis section:

*A power analysis was conducted using R studio (package pwr) (RStudio, 2020) based on detecting a medium effect size for our main hypothesis concerning differences in attractiveness and weight judgments in paired samples t-tests (as shown in previous studies comparing attractiveness and weight ratings (Carey et al., 2019)). This suggested that the first three experiments would need at least 33 participants (power = 0.80, alpha of .050, d = 0.50). However, for each experiment we over recruited to allow for attrition and correlational analysis for which we had no a priori effect size.*

The below extract is taken from a passage in the discussion considering these issues around power:

*As mentioned in the data analysis section, we recruited more participants than needed in anticipation of attrition. However, although we recruited enough participants to be somewhat over-powered to detect effects in the pairwise comparisons, we were not sufficiently powered in terms of detecting correlations, particularly for correlations with WHR. For WHR, we were only powered enough to detect large directional relationships (r > 0.71), and for correlations with EDE-Q scores we could detect correlations at r >0 .29 for experiment one, r > .34 for experiment two, and r > .37 for experiment three. This may go some way to explaining why we did not detect the relationships we anticipated around WHR and attractiveness judgements, and why we did not detect relationships between aesthetic judgements and some of the EDE-Q subscale scores. Future investigations into these relationships should ensure that sufficient participants are recruited to detect even small effects, given the complex nature of the phenomena in question.*

**Figure 1: I would suggest re-ordering the four pictures according to the order they are mentioned in the manuscript. Also, referring to the upper/lower part of the Figure 1 in the corresponding task may be more incisive.**

We have adapted the figure and made reference to the specific part of the figure in the corresponding task.

*Figure 1*

*Example stimuli: Selfie (top left), allocentric (top right), egocentric (bottom left), and selfie-stick (bottom right) perspectives.*

**Line 297: I suggest reporting the analyses concerning the experimental task (i.e., attractiveness/weight judgments) first and then (e.g., before line 307) those relative to the EDE-Q: this order seems more congruent with the timing of tasks/questionnaire administrations and with the “hierarchy” study aims (i.e., as I understand the core aim is to compare judgments relative to different perspective, while the possible correlation with eating disorders is secondary).**

We have re-structured the results section of each experiment based on these helpful recommendations. We first address the attractiveness and weight judgements, then outline WHR results, and state EDE-Q related results at the end of the section.

**Also, as previously mentioned, I would report the analyses (which they seem the same in experiment one-to-three, before introducing each single experiment).**

This comment helped us to restructure the data analysis section of the manuscript so that it is clearer and easier to follow. We now include a data analysis section in the ‘General Methods’, which outlines the common aspects of the analyses across experiments. Then in each individual methods section we outline the specific details of the data analysis of that section. An example from experiment one is provided below, alongside the general data analysis section.

*Data Analysis (General)*

*Firstly, in experiments one to three we calculated the EDE-Q subscale scores for each participant by taking the mean of the relevant items for each subscale (Carey et al. 2019). We treat the EDE-Q scores as ordinal data, as is the norm in the ED literature (Wu & Leung, 2017; Jennings & Phillips, 2017.) We then calculated the separate mean scores for weight and attractiveness ratings for each image within each perspective. This is calculated by taking the average rating for each stimuli across participant responses, for both weight and attractiveness ratings, so each stimuli had an average weight and attractiveness score across participants. We used paired samples t-tests (or their non-parametric equivalents) to calculate whether the differences in aesthetic ratings were different across perspectives. When calculating normality, we expect that EDE-Q scores will be non-normal, based on previous studies using a similar demographic. For this reason, we do not analyse outliers.*

*We directly tested the hypothesis that aesthetic and weight judgements will be related to eating disorder symptomatology by assessing if there were significant correlations between EDE-Q scores and the differences in attractiveness and weight judgements between selfies and the other (comparison) perspectives. To this aim, we first calculated difference scores in attractiveness and weight ratings between the two image types.*

*A power analysis was conducted using R studio (package pwr) (RStudio, 2020) based on detecting a medium effect size in paired samples t-tests (as shown in previous studies comparing attractiveness and weight ratings (Carey et al., 2019)). This suggested that the first three experiments would need at least 33 participants (power = 0.80, alpha of .050, d = 0.50).*

*We took waist and hip measurements in each photo to calculate WHR. Waist measurements were identified as the width across the body at the smallest visible point of the lower torso. Hip measurements were identified as the widest point of the lower torso below the waist at the top of the thighs. We then tested the hypothesis that WHR will be related to attractiveness judgements, by assessing correlations between differences in WHR and differences in attractiveness judgements across perspectives. Specific details on this process can be found in each experiment’s data analysis section.*

Below is an example of the shortened data analysis section for experiment one, which includes only the specific details that are different for each individual experiment:

*Data Analysis (Experiment One)*

*We followed the analysis steps laid out above. To directly test hypotheses that selfies will be judged as more attractive and slimmer than allocentric images, we compared the stimuli across perspectives on attractiveness and weight ratings using pairwise comparisons. If data were normally distributed, we used paired samples t-tests and Pearson’s r correlations. If data were not normally distributed, we used Wilcoxon signed rank tests and Kendall’s tau correlations. Bonferroni correction was used for multiple comparisons.*

*Attractiveness difference scores were calculated by subtracting attractiveness scores for allocentric images from selfie images such that positive scores represented greater attractiveness judgements for selfie images and negative scores represented greater attractiveness for allocentric images. The reverse calculation was done for the weight rating such that positive scores represent slimmer judgements for selfies and negative scores represent slimmer scores for allocentric images.*

*To examine the relationships between WHR and attractiveness ratings we subtracted WHR for allocentric images from selfie images and correlated these with the attractiveness differences as calculated above. Because the majority of the bodies in our stimuli had a WHR > .07 and we anticipate that selfies would be associated with more optimal WHR, negative correlations between attractiveness and WHR differences would represent relationships in the predicted direction (greater attractiveness would be associated with WHR’s closer to optimal in selfie images).*

**Anyway, concerning correlational analyses, I am not confident that normality should be checked for EDE-Q scores given the ordinal nature of Likert-scores. This is, indeed, only a matter of writing since the Authors used non-parametric correlational analyses.**

Many thanks for highlighting that we had not clarified how we were treating the EDE-Q scores. We have added a passage around this to the manuscript.

*We treat the EDE-Q scores as ordinal data, as is the norm in the ED literature (Wu & Leung, 2017; Jennings & Phillips, 2017.) In line with other studies in the field, we checked EDE-Q scores for normality.*

**In this regard, did the Authors checked for the actual achieved power of the correlational analyses? Since sample size was computed on pairwise comparisons, correlations may/may not be enough powered, possibly explaining why the Authors did not find certain expected results. The Authors may then consider to discuss this point among limitations.**

We had not checked actual achieved power of correlational analyses, and we are grateful to the reviewer for this very useful suggestion. We have checked the power achieved in the analyses and reflected on this within the discussion.

*Although we recruited enough participants to be sufficiently powered to detect effects in the pairwise comparisons, we were not sufficiently powered in terms of detecting correlations, particularly for correlations with WHR. For WHR, due to limitations concerning the number of stimuli, we were only powered enough to detect large directional relationships (r > 0.71), and for correlations with EDE-Q scores we could detect correlations at r >0 .29 for experiment one, r > .34 for experiment two, and r > .37 for experiment three (all calculations were made using the R studio pwr package (RStudio Team, 2020). This may go some way to explaining why we did not detect the relationships we anticipated around WHR and attractiveness judgements, and why we did not detect relationships between aesthetic judgements and some of the EDE-Q subscale scores. However, differences that were observed between the pattern of results do at least reflect a difference in magnitude of effect between conditions. In addition, for many of our null correlations the coefficients were close to zero, particularly concerning potential relationships between the EDE-Q and differences between selfie vs. egocentric images. Future investigations into these relationships should ensure that sufficient participants are recruited to detect even small effects, given the complex nature of the phenomena in question.*

**Similarly, did the Authors considered/analysed outliers? It is quite interesting that dependent variables in experiment one-to-three were all non-normal: outliers may be a related issue.**

We did not analyse outliers in this set of studies, as in samples completing the EDE-Q alongside other measures are often non-normal, and we expect to see outliers in the data given the nature of the phenomena explored. However, we had not made this sufficiently clear in the manuscript, so based on this useful suggestion we have added a section explaining this.

*When calculating normality, we expect that EDE-Q scores and accompanying responses will be non-normal, based on previous studies using a similar demographic. We also implemented non-parametric statistics to reduce the impact of skewed data. For this reason, we do not analyse outliers.*

**Additionally, Authors may consider providing boxplots with participants single data point for each experiment, this may help them detecting outliers/extreme values and the reader to acknowledge data distribution.**

Based on the reviewer’s suggestion here, we have provided violin plots with overlaid box plots and data points for each experiment to demonstrate the distribution of the data. We hope that this is useful in interpreting the results and highlights the distribution. These can be found on pages 17, 25, 32, 39, and 40.

**Please, clarify how the dependent variable was computed. This wording suggests that the Authors got ten measures of weight/attractiveness relative to each perspective: were then these measures collapsed (how?) into one variable (one for weight/attractiveness) in each perspective?**

It was clear we had not sufficiently explained how the dependent variable was computed in each experiment. We have added some clarification around this to the data analysis section, and hope that this process is now clearer and easier to follow.

*We then calculated the separate mean scores for weight and attractiveness ratings for each image within each perspective. This is calculated by taking the mean rating for each stimuli across participant responses, for both weight and attractiveness ratings, so each stimuli had an average weight and attractiveness score across participants.*

**Furthermore, the Authors may make explicit the purpose of parametric/non-parametric analyses: they reported that paired-sample t-tests/ Wilcoxon tests were used, but it is not clear to which purpose. I imagine the goal was comparing weight/attractiveness between the two perspective in each experiment: is that possible that this is indeed reported below at line 302-205? If this is the case, I strongly suggest moving this part before.**

Many thanks for this helpful suggestion, we have re-structured this passage to make it clearer what the purpose of the t-tests (or non-parametric equivalents) were.

*We used paired samples t-tests (or their non-parametric equivalents) to calculate whether the differences in aesthetic ratings were different across perspectives.*

**Additionally, if the Authors would accomplish my suggestions of reporting common analyses before, here (and after) they might only report the hypothesis tested in this experiment (e.g., lines 302-303).**

We found that revising the manuscript in line with this suggestion improved the clarity and flow of the data analysis section. We re-drafted this section, as above, to include the common analyses in a general data analysis section, and then specified the aspects that were unique to each experiment in their respective sub-sections.

**I was wondering why the Authors collected participants’ BMI, although it seems reasonable. Is this to ensure that participants were not overweight/underweight? If so, why this should be considered relative to the study purposes? Did the Authors compared participants’ BMI and models’ BMI?**

It is standard practice to calculate BMI when collecting EDE-Q responses, so we collected this to be able to describe participants. We have clarified this in the appropriate section of the manuscript.

*Participants then completed the EDE-Q and recorded their own self-reported weight and height (to calculate BMI) before finishing the questionnaire and debrief. BMI was calculated to describe participants.*

We have also added a section around BMI to the discussion section of the manuscript.

*We collected information on participant weight and height to calculate BMI, which is standard practice when asking participants to respond to EDE-Q questions. Some participants had BMI that would be categorised as underweight. We did not incorporate this information into analysis, but future research may wish to consider the effect of participant BMI when making aesthetic judgements of social media images, particularly relating to our hypothesis that those who are already more vulnerable to disordered eating thoughts and behaviours might be more negatively affected by this kind of content.*

**Also, it may be useful and more informative to report the perspective compared near the number of the experiment.**

We found this suggestion very helpful, and have implemented in throughout the manuscript.

**I wonder whether the Authors could support the hypothesis of a “bias against egocentric pictures” somehow with their results (please considered this more a curiosity, rather than a critical point).**

Based on this suggestion we have provided more information around the hypothesis of a bias against egocentric pictures. Here we outline how the association between the egocentric image and the self (as an egocentric image is taken from the perspective of somebody looking down at their own body), in combination with the tendency to view one’s own body negatively, may lead to a bias against egocentric images in terms of attractiveness judgements.

*Thus, we speculate that these results are not primarily driven from a preference for selfies, but from a bias against egocentric images. Egocentric images are taken from the perspective of somebody looking down at their own body, which may therefore be associated more strongly with the self, compared to the selfie images of the same bodies. Based on this, a preference for the selfies compared to egocentric images may be in part driven by the negative bias towards the own body that has been found in female participants (Dijkstra & Barelds, 2011). If the egocentric image is associated with the self, participants may have applied this bias and thus judged these images as less slim and attractive. Thus, the higher attractiveness and lower weight ratings for selfies may be due to egocentric images being judged as larger and less attractive. This may also go some way to explaining why selfies were not judged to be more attractive than allocentric images, despite being judged as slimmer. An allocentric image may not tap into the self-bias in the same way that an egocentric image does, thus results might be based on a bias against the egocentric images.*

**For instance, I would be curious to know whether the BMI was associated with the ratings of the egocentric pictures/or the preference for selfies (especially considering that there was no -the expected - correlation with eating disorders measures). In this regard, the Authors may consider that BMI seems associated to body size distorted judgments (e.g., see Tagini et al., 2021 https://doi.org/10.1007/s00221- 021-06215-4***)*

Many thanks for highlighting this useful reference. We have incorporated this into a section in the discussion of the revised manuscript regarding possible future research directions.

*Furthermore, we did not consider whether participant BMI was associated with aesthetic judgements, particularly in terms of egocentric images and the preferences for selfies. Meta-analysis indicates that participants in larger bodies are less accurate in body size estimations, which may be due to body dissatisfaction or different somatosensation (Tagini, Scarpina & Zampini, 2021.) Based on this, future research might consider how body size of the observer influences aesthetic judgements.*

**Overall, since many results are reported, I believe that summarizing tables may efficaciously help the reader to compare results across studies, at once.**

Based on this suggestion we have incorporated a table summarizing the results into each of experiments one to three.

**As already mentioned, I suggest reporting sample size calculation after the analyses section since expected power is computed accordingly.**

We have added the power analysis to the end of the analysis section in line with the reviewer’s helpful suggestion (see above).

**In** **this regard, please specified that one-way “repeated measures” ANOVAs were used.**

We have added this information to the revised manuscript (page 34.)

**Could the Authors clarify how many trials were included in each block (please, also in the previous experiments)?**

Many thanks for highlighting that we had not included this information; we have now added it to both the general methods section and the methods section for experiment four.

*Participants saw each image once, thus viewing ten images in each condition/block, 40 trials overall for experiments one to three. In experiment four, participants were shown all images across each condition and were asked to make aesthetic judgements of each one (80 trials overall.)*

**Was each combination of model/perspective repeated only one time (10 trials in each perspective)?**

Each combination was presented twice to the participant, once to judge attractiveness and the other to judge weight. We hope that we have made this clearer in the revised manuscript, based on the reviewer’s useful feedback.

**Could the Authors specify why the Bayes factor was computed only in experiment four?**

We have incorporated further information on why we only computed BFs in experiment four in the data analysis section.

*As this experiment was designed to replicate our previous findings from experiments one to three, we decided to include Bayesian analysis for all non-significant results to determine the likelihood of the data occurring under the null hypothesis (Kass & Raftery, 1995). The BF represents a likelihood ratio of the alternative relative to the null hypothesis (*[*Dienes, 2014*](https://www.sciencedirect.com/science/article/pii/S0022096522001060#b0085)*). A BF > 3 indicates evidence for the alternative hypothesis, a BF < 0.33 indicates evidence for the null hypothesis and a BF between 0.33 and 3 being inconclusive (*[*Dienes, 2014*](https://www.sciencedirect.com/science/article/pii/S0022096522001060#b0085)*).*

**I believe the figure here is in the wrong place.**

Many thanks for highlighting our error, we have moved the figure to the correct position in the revised manuscript, on page 41.

**I found this point on the possible effect of personality traits associated to selfies a bit confusing. It seems that selfies are related to both “good” and “bad” attributes but it is not clear how these sides related to the study results, respectively. I would clarify the wording and the hypothesis proposed: indeed, it is quite interesting (and reasonable) that weight judgments rely more on perceptive bottom-up processing of visual information whereas attractiveness judgments may likely include also topdown components.**

We have clarified both the wording and hypothesis proposed, and have also attempted to integrate the reviewer’s interesting suggestion here regarding possible processes.

*It may also be that participants viewed selfie images as being more attractive due to the qualities that they convey or are associated with. Selfies have been suggested to endorse the idea that the person in the photo is more extroverted, sociable, and open to experience; qualities that have previously been associated with attractiveness (Segal-Caspi, Roccas & Sagiv, 2012; Musil et al., 2017). However, selfies have also been linked to more negative personality traits; such as narcissism and untrustworthiness (Taylor, Hinck & Lim, 2017; Kramer et al., 2017), which may partly dissociate a link between slimness and attractiveness. Interestingly, significant difference in slimness between perspectives did not always have a corresponding significant difference in attractiveness ratings in these experiments. This may suggest that the link between slimness and attractiveness is not as clear as anticipated, and other factors, such as assumed personality traits and related judgements, may play some role in these aesthetic values. It may be, for example, that weight judgements are predicated more on bottom-up perceptual processes, and attractiveness judgements are linked to top-down components too. Further research can consider these questions in greater depth.*

**Did the Authors try to correlate slimness and attractiveness judgments in each perspective?**

We did not analyse the correlations between slimness and attractiveness in each perspective, as we felt this was beyond the scope of the current study, and that the manuscript was already quite long! We have referred to this in the discussion of the revised manuscript:

*We did not analyse correlations between slimness and attractiveness judgements, as this was beyond the scope of the current study, which aimed to consider differences in aesthetic judgements across perspectives. However, in the future researchers may want to explore whether or not the differences in attractiveness judgements are driven by perceived slimness, or another contributing factor.*

**Finally, I suggest a careful check for typos.**

Many thanks for highlighting that a thorough proof-read of the article was needed. We have carefully checked over the revised manuscript.
